# Aurintricarboxylic acid structure modifications lead to reduction of inhibitory properties against virulence factor YopH and higher cytotoxicity

**DOI:** 10.1007/s11274-016-2123-3

**Published:** 2016-08-25

**Authors:** Alicja Kuban-Jankowska, Kamlesh K. Sahu, Magdalena Gorska, Pawel Niedzialkowski, Jack A. Tuszynski, Tadeusz Ossowski, Michal Wozniak

**Affiliations:** 1 Department of Medical Chemistry, Medical University of Gdansk, Gdańsk, Poland; 2 Department of Medical Microbiology and Immunology, University of Alberta, Edmonton, Canada; 3 Department of Physics, University of Alberta, Edmonton, Canada; 4 Department of Analytical Chemistry, University of Gdansk, Gdańsk, Poland

**Keywords:** Aurintricarboxylic acid (ATA), Eriochrome cyanine R, Pararosaniline, YopH, Protein tyrosine phosphatases (PTPs)

## Abstract

*Yersinia* sp. bacteria owe their viability and pathogenic virulence to the YopH factor, which is a highly active bacterial protein tyrosine phosphatase. Inhibition of YopH phosphatase results in the lack of *Yersinia* sp. pathogenicity. We have previously described that aurintricarboxylic acid inhibits the activity of YopH at nanomolar concentrations and represents a unique mechanism of YopH inactivation due to a redox process. This work is a continuation of our previous studies. Here we show that modifications of the structure of aurintricarboxylic acid reduce the ability to inactivate YopH and lead to higher cytotoxicity. In the present paper we examine the inhibitory properties of aurintricarboxylic acid analogues, such as eriochrome cyanine R (ECR) and pararosaniline. Computational docking studies we report here indicate that ATA analogues are not precluded to bind in the YopH active site and in all obtained binding conformations ECR and pararosaniline bind to YopH active site. The free binding energy calculations show that ECR has a stronger binding affinity to YopH than pararosaniline, which was confirmed by experimental YopH enzymatic activity studies. We found that ATA analogues can reversibly reduce the enzymatic activity of YopH, but possess weaker inhibitory properties than ATA. The ATA analogues induced inactivation of YopH is probably due to oxidative mechanism, as pretreatment with catalase prevents from inhibition. We also found that ATA analogues significantly decrease the viability of macrophage cells, especially pararosaniline, while ATA reveals only slight effect on cell viability.

## Introduction

*Yersinia* genius represents the species of bacteria pathogenic to humans, plague-causing *Yersinia pestis* which is one of the most virulent infectious agents threatening humans, *Yersinia pseudotuberculosis* inducing tuberculosis-like symptoms and septicemia or *Yersinia enterocolitica* responsible for gastrointestinal disorders (Trosky et al. 2008). There are still many human cases caused by *Y. pestis*, with the greatest frequency of human plague infections occured in Africa (Stenseth et al. 2008; Respicio-Kingry et al. 2016). Moreover, the presence of *Y. pestis* in wild reservoir animals (i.a. from national parks) is detected also in highly developed countries (Mize and Britten 2016). *Y. pestis* is transmitted through blood by fleas from its natural reservoirs, mainly rodents, squirrels, chipmunks or rabbits, and leads to the bubonic form of plague (Achtman et al. 2004). The inhalation of the infectious respiratory droplets of bacteria *Y. pestis* results in the most severe primary pneumonic plague, with mortality rates approaching 100 percent in the absence of treatment (Pechous et al. 2016). Both forms can lead to infection of the blood, causing bacteremia and septicemic plague.

Infection caused by *Y. pseudotuberculosis* and *Y. enterocolitica* may occur via consumption of contaminated milk-derived products, vegetables or meat. *Yersinia* are the third cause of bacterial diarrhea in Europe. The enteric yersiniosis caused by *Y. enterocolitica* manifests with diarrhea, fever, abdominal pain, and in rare cases systemic forms can be observed (Le Guern et al. 2016).

During infection, those three species of *Yersinia* bacteria translocate virulence effectors (Yops) into a host cell due to type III secretion system (Atkinson and Williams 2016; Bahta and Burke 2012) which leads to inhibition of the innate immune response (Schwiesow et al. 2015; Viboud et al. 2003). YopH protein tyrosine phosphatase is one of the effectors, which causes blockage of phagocytosis (Deleuil et al. 2003) by dephosphorylation of the focal adhesion kinase (FAK) and suppression of the reactive oxygen species production by macrophages (Trulzsch et al. 2008).

The YopH phosphatase is similar to eukaryotic PTPs and contains a catalytic cysteine residue in the active site, which is essential for enzymatic activity, as it plays function of a nucleophile in catalytic process (Black et al. 2000). The catalytic cysteine exists in a thiolate anion form and is highly vulnerable to oxidation. The cysteine residue in the active site determines the enzyme activity only in the non-oxidized state, therefore its oxidation leads to inactivation of the enzyme. Depends on the oxidation state, the sulfenic, sulfinic or sulfonic acid can be formed (Ostman et al. 2011).

The risk of utilizing of *Yersinia* by unauthorized groups as a biological weapon of terror (Pechous et al. 2016; Hawley and Eitzen 2001), the climate change increasing the risk of plague outbreaks (Ben-Ari et al. 2011), as well as the growing resistance of humans to antibiotics, are the reasons to search for new treatment options. The virulence factor YopH is a perfect candidate for a new drug target as it is essential for virulency of *Yersinia* bacteria (Bohmer et al. 2012; Liang et al. 2003).

**Fig. 1:**
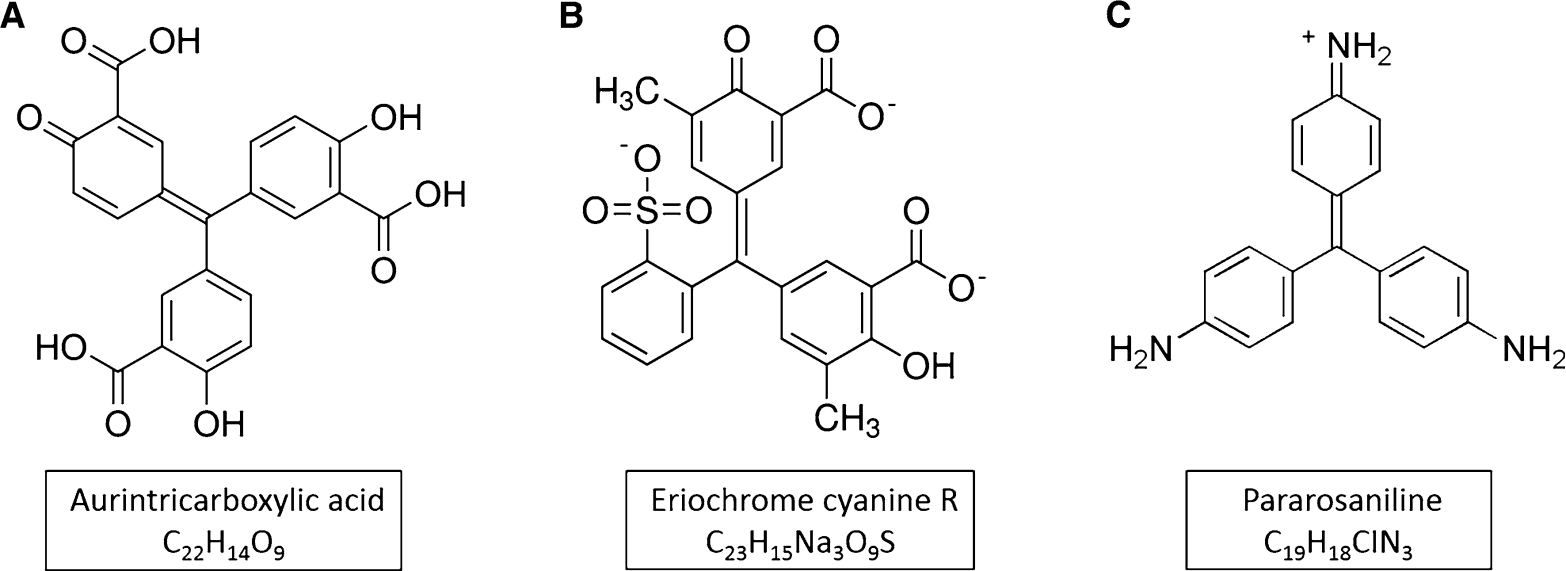
The structure of aurintricarboxylic acid (**a**) and its analogues: eriochrome cyanine R (**b**) and pararosaniline (**c**)

The numerous YopH inhibitors were reported to inhibit YopH activity, mostly at micromolar concentrations (Heneberg 2012), such as salicylic acid derivatives (Huang et al. 2010), natural substrate mimetics, compounds with carboxyl groups (Zhang 2003), as well as natural compounds, such as bromotyrosine alkaloids purified from a marine sponge (Yin et al. 2011) or chicoric acid (Kuban-Jankowska et al. 2016). The most effective YopH inhibitor characterized to date is aurintricarboxylic acid (Fig. 1a), with an IC_50_ values in nanomolar ranges, discovered by Liang et al. 2003, and confirmed by our studies (Kuban-Jankowska et al. 2015).

Aurintricarboxylic acid is a polyaromatic carboxylic acid derivative with a continuously growing number of biological activities in which it participates. ATA is known to possess anti-viral and anti-bacterial properties by inhibiting many enzymes needed for cell and virus replication, such as polymerases, helicases, nucleases, topoisomerases and bacterial protein tyrosine phosphatases (Bardhan et al. 2011; Myskiw et al. 2007). It has been discovered that ATA inhibits the influenza-A virus by reducing its ability to reproduce in cultured post-infected canine kidney cells. The reduction of viral reproduction and infection was observed only when ATA was applied after infection, and not after only pretreatment before infection (Hashem et al. 2009).

ATA or its analogues are seriously considered as drug candidates in drug design studies for infectious diseases, including epidemics caused by *Yersinia* sp. We have previously described the mechanism of aurintricarboxylic acid caused inhibition of YopH. In the present study we examine the inhibitory properties of aurintricarboxylic acid analogues, such as eriochrome cyanine R and pararosaniline, to evaluate the effect of structure modifications on inhibitory properties of the compound.

## Methods and materials

### Docking studies

The initial structure of YopH was imported from the RCSB protein data bank (www.pdb.org) with code 2YDU.pdb (Kim et al. 2011). The structure was minimized using taff.ff force field of the Molecular Operating Environment software (MOE, Chemical Computing Group). Chain A of this pdb file contains 306 residues. The ligand was removed from this pdb file and ATA analogues were docked into the structure of YopH. A blind flexible docking simulation was performed, where the binding site was assumed to be the entire protein. The side chains were kept free to move during force field refinement. Alpha PMI is the placement method used with default settings (sample per conformation = 10, maximum poses = 250). London dG rescoring was used with Alpha PMI placement. Termination criteria for force field refinement were set as gradient = 0.001 and iterations = 500.

### Molecular dynamics simulations

Top scoring poses from docking that interacted with Cys403 were retained for molecular dynamics simulations using amber12 software. We allowed the Leap module of Amber (Case et al. 2005) to add missing hydrogen atoms and heavy atoms using the Amber force field (ff10) parameters (Lindorff-Larsen et al. 2010). To neutralize the charge of the system, we added sodium/chloride ions. The model was immersed in a truncated cubical shell of TIP3P water (Jorgensen et al. 1983). A time step of 2 fs and a direct-space non-bonded cutoff of 10 Å were used. After the protein preparation, all systems were minimized to remove the steric clashes that occurred. The systems were then gradually heated to 300 K over a period of 50 ps and then maintained in the isothermal–isobaric ensemble (NPT) at a target temperature of 300 K and a target pressure of 1 bar using a Langevin thermostat (Izaguirre et al. 2001) and a Berendsen barostat with a collision frequency of 2 ps and a pressure relaxation time of 1 ps, respectively (Berendsen et al. 1984). We constrained hydrogen bonds using the SHAKE algorithm (Ryckaert et al. 1977). We have used the velocity-Verlet algorithm (default algorithm for the Amber MD package) for MD simulations. Particle mesh Ewald (PME) procedure was used to treat long-range electrostatic interactions using default parameters (Darden et al. 1993). After bringing the systems to our suitable temperature and pressure of 300 K and 1 bar, respectively and equilibrating the system for 500 ps, the production run was continued for 20 ns in the isothermal–isobaric ensemble at the target temperature of 300 K and target pressure of 1 bar using the same Langevin thermostat and Berendsen barostat. The structures in the trajectories were collected at 10 ps intervals. The analysis of trajectories was performed with the Ptraj module of Amber.

### Binding affinity calculations

For the binding free energy calculations, we used the standard MM/GBSA method and MMPBSA.py python script (Miller et al. 2012). Before the MM/GBSA analysis, all water molecules and the sodium ions were excluded from the trajectory. The dielectric constant used for the solute and surrounding solvent was 1 and 80, respectively. During the analysis of the MM/GBSA trajectory, snapshots were gathered at 10 ps intervals from the last 500 ps of the 20 ns trajectory.

### YopH activity assay

Eriochrome cyanine R and pararosaniline were obtained from Merck Calbiochem. The bacterial recombinant YopH protein tyrosine phosphatase from *Y. enterocolitica* expressed in *Escherichia coli* was from Sigma Aldrich (Product Nr Y 4127). The solution of the recombinant PTP YopH was prepared in 10 mM HEPES buffer pH 7.4. The final concentration of phosphatase in reaction samples was 0.25 μg/mL (3 nM). The YopH enzyme was untreated (control) or treated with solution of ATA analogues. The ATA analogues were prepared in water in a range of final concentrations: from 100 nM to 1 mM in incubation samples. The assay was performed in 96-well microplates, and the final volume of each sample was 200 μL. After 15 min incubation with inhibitors in 37 °C the enzymatic activity of YopH was measured using 0.5–10 mM chromogenic substrate *para*-nitrophenyl phosphate (*p*NPP) in 10 mM HEPES buffer pH 7.4, at 37 °C. Phosphatase hydrolyzed *p*NPP to *para*-nitrophenol and inorganic phosphate. *Para*-nitrophenol is an intensely yellow colored soluble product under alkaline conditions. The increase in absorbance (due to *para*-nitrophenol formation) is linearly proportional to enzymatic activity concentration (with excessive substrate, i.e. zero-order kinetics) and was assessed at 405 nm on a microplate reader Jupiter (Biogenet) using DigiRead Communication Software (Asys Hitech GmbH). The reversibility of the ATA analogues induced inactivation was determined with the reduction assay by 30 min incubation with 10 mM dithiothreitol (DTT). Additionally, the activity of YopH after treatment with 50 µM ATA analogues was measured in presence and in absence of catalase (0.3 mg/mL).

### Cell culture

The mouse macrophage cell line J774.2 was obtained from European Collection of Cell Culture (ECACC, UK). The cells were cultured in DMEM medium supplemented with 10 % fetal bovine serum, 100 μg/mL penicillin/streptomycin and 2 mM l-glutamine. The culture was maintained at 37 °C and in an atmosphere containing 5 % CO_2_. DMEM medium and supplements were obtained from Sigma Aldrich. The cell culture density was kept to maximum 1 × 10^6^ cells/mL. At least every 2 days the medium was replaced with the fresh one, and the cells were counted and reseeded to maintain the recommended density.

### Cell viability assay (MTT assay)

The cells (1 × 10^6^ cells/mL) were untreated (control) or treated with solution of ATA and ATA analogues dissolved in water. The final concentrations of the compounds in cell plates were 100 nM, 10 µM, 100 µM. After the appropriate incubation time (24 and 72 h) were suspended in solution of 0.5 mg/mL MTT(3-[4,5-dimethylthiazol-2-yl]-2,5-diphenyltetrazolium bromide) in DMEM without phenol red. The 100 µL samples were incubated for 2–4 h at 37 °C in 96-well plates. When the purple precipitate was clearly visible under the microscope, 100 µL of DMSO was added to each well and the plate with cover was left in the dark for 2–4 h. The absorbance at 540 nm was determined using a microplate reader.

### Statistical analysis

The experiments were performed at least three times. The data were applied and analyzed with GraphPad Prism (GraphPad Software v.4). Statistical analyses were performed using ANOVA combined with Tukey’s test or *T*-test combined with Wilcoxon test. The data were expressed as mean ± SD. Differences between means were considered significant for *p* < 0.05.

## Results

### Docking studies of ATA analogues for YopH binding site

**Fig. 2:**
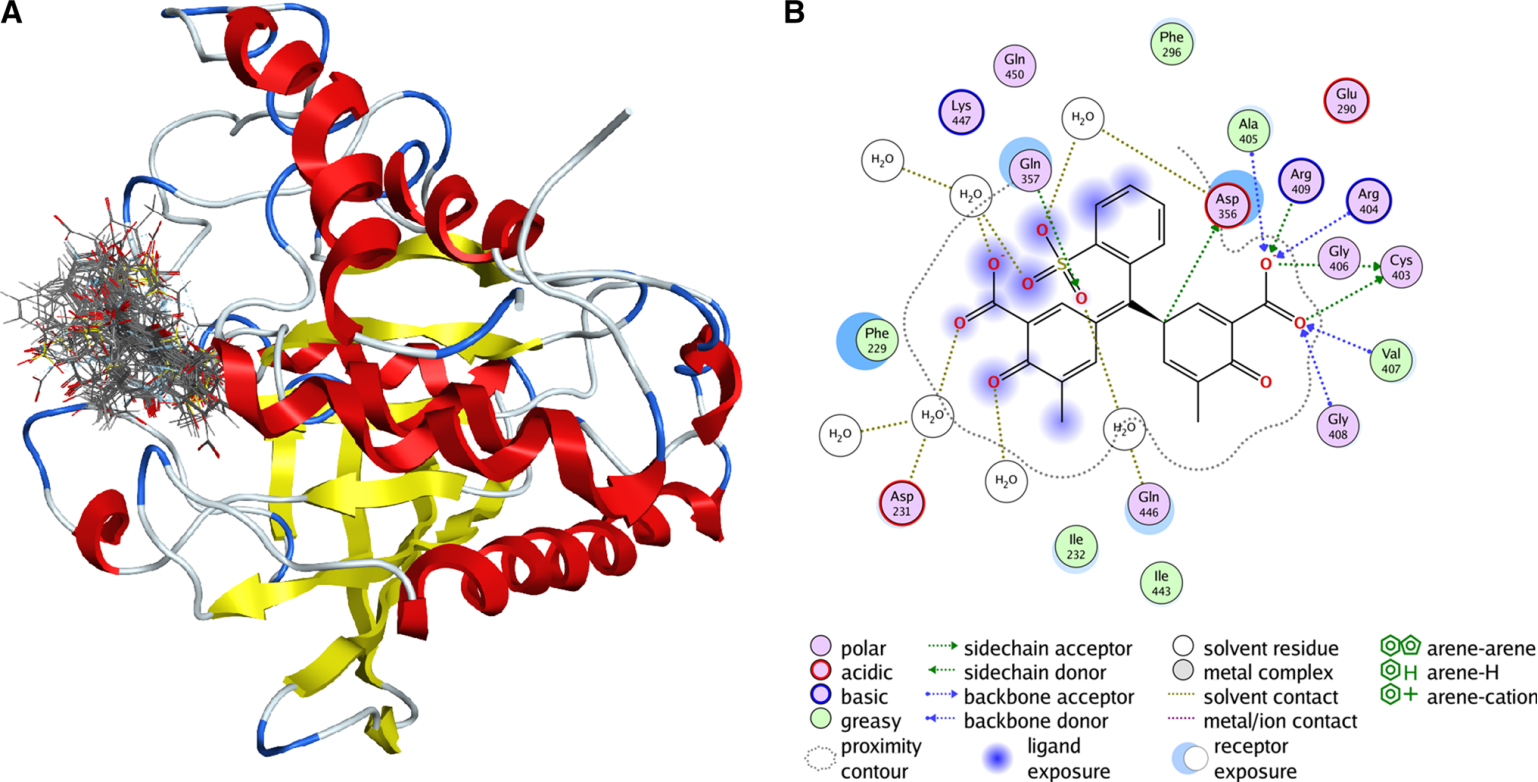
Computational analysis of eriochrome cyanine R binding and interactions in YopH active site. **a** The site of binding for top 30 conformations of eriochrome cyanine R obtained from docking of ECR into the YopH structure. In each conformation ECR binds in the YopH active site. **b** The PLIF diagram for the best binding pose of ATA in the YopH binding site. In predicted binding pose, the carboxyl group of ECR is directed toward essential Cys403, Arg409 and Asp356 residues in the active site. There are electrostatic interactions between polar groups of ECR with Cys403, Arg409 and Asp356

**Fig. 3:**
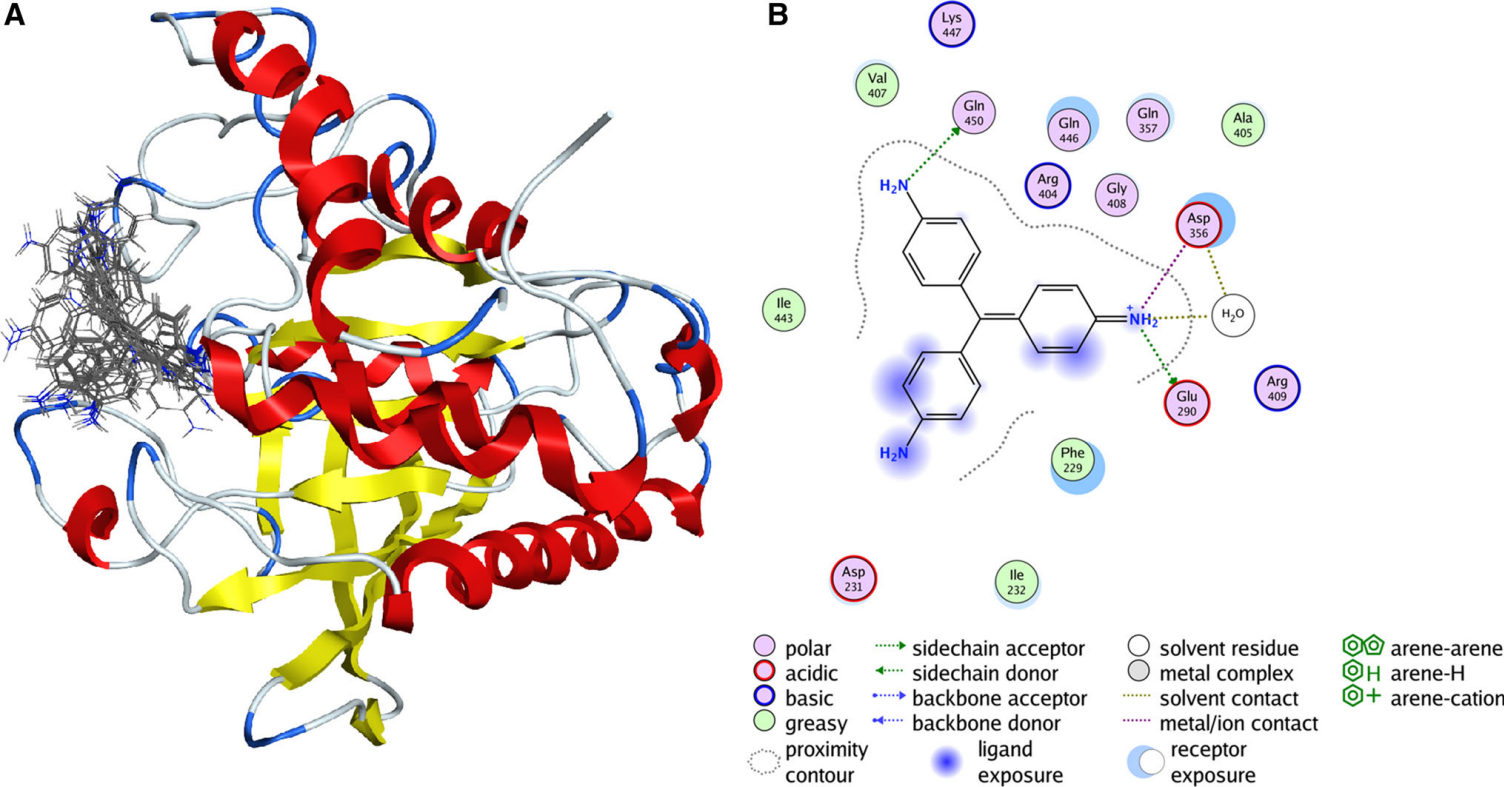
Computational analysis of binding and interactions of pararosaniline in YopH active site. **a** The site of binding for top 30 conformations of pararosaniline obtained from docking of pararosaniline into the YopH structure. In each conformation, pararosaniline binds in the YopH active site. **b** The PLIF diagram for the best binding pose of pararosaniline in the YopH binding site. In predicted binding pose, there are interactions with essential Asp356

Eriochrome cyanine R and pararosaniline molecules were docked into the 3D structure of YopH in order to investigate the possible binding conformation and affinity. We performed blind flexible docking and retained top 30 conformations from docking runs. In all 30 conformations ATA analogues are bound to the active site of YopH, as shown in Fig. 2a for ECR and Fig. 3a for pararosaniline. The docking studies showed that ATA analogues can be easily accommodated inside the binding site and binds specifically in a catalytic center of YopH, in a similar manner to that of the natural substrate, phosphotyrosine (Figs. 2a, 3a).

### The molecular dynamics simulations of YopH with ATA analogues

To study the binding conformation of ATA analogues in the YopH active site we performed molecular dynamics simulations using Amber12 and identified top scoring poses from docking studies. The interactions of ATA analogues in the YopH binding site are presented as a PLIF diagram (protein ligand interaction fingerprints) in Figs. 2b and 3b. The dotted line around the molecule shows solvent contact and dotted arrows represent hydrogen bonds between amino acid residues from YopH and ATA analogues. In the predicted binding pose, the carboxyl groups of ECR is directed toward essential Cys403 and Arg409 residues in the YopH active site (Fig. 2b). Under such steric conditions there is likelihood of hydrogen bond formation between the arginine residue and carboxyl groups of ECR (Fig. 2b). The positively charged arginine residue of the YopH active site is likely to attract the negatively charged carboxyl groups from ECR. As shown in the PLIF diagram, pararosaniline is able to utilize its polar groups to interact electrostatically with Asp356, general acid in catalytic mechanism of YopH, essential for its enzymatic activity (Fig. 3b). In addition to this, NH2 groups of pararosaniline also interact electrostatically with Asp231, Glu290 and Gln450.

### The binding energies of ATA analogues complexes with YopH

**Fig. 4:**
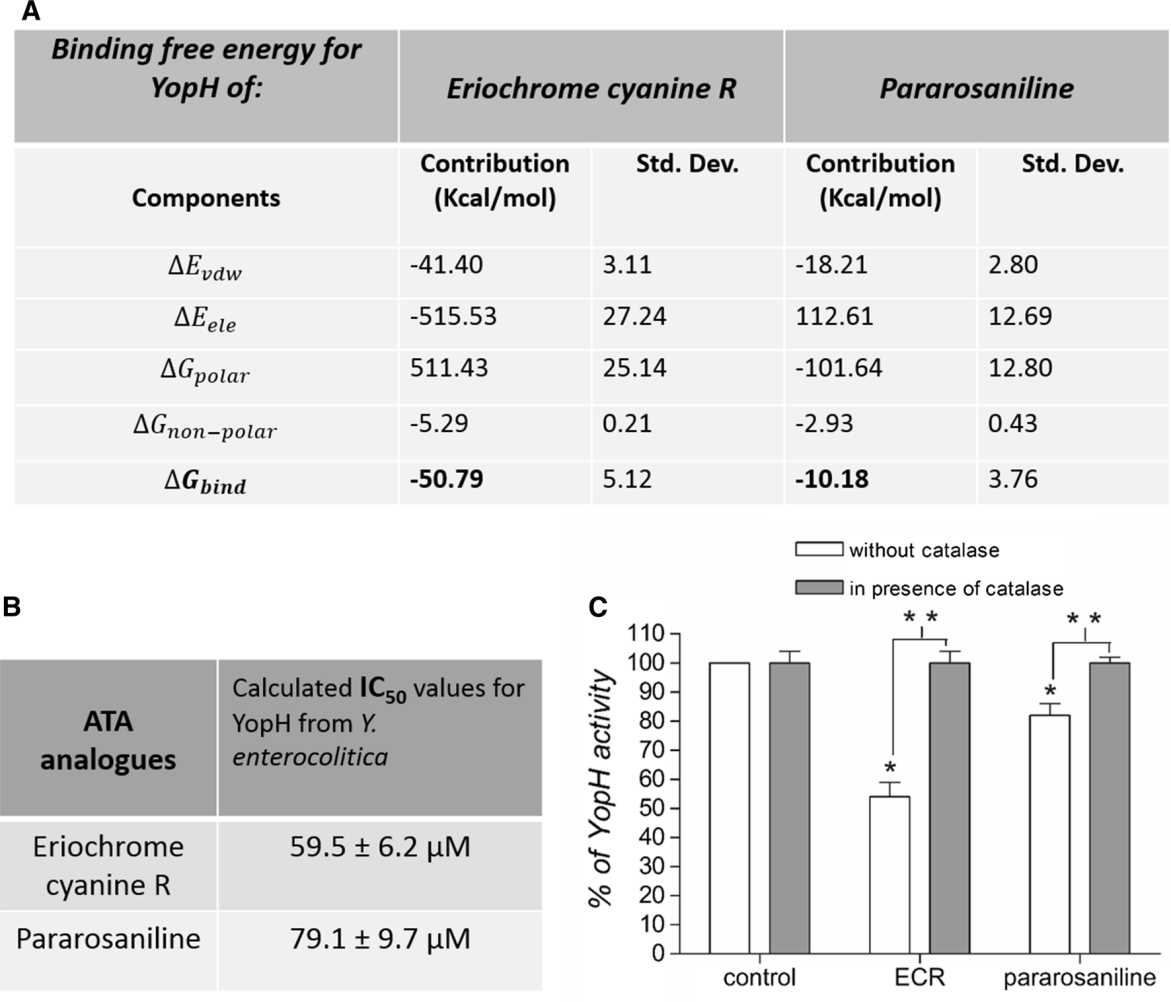
The binding affinity and inhibitory properties of ATA analogues: eriochrome cyanine R and pararosaniline to YopH. **a** The binding affinity of ECR and pararosaniline to YopH active site presented as binding free energy and its components for the YopH-ECR, and YopH-pararosaniline complexes by MM/GBSA methods (kcal/mol). **b** The inhibitory properties of ECR and pararosaniline to YopH presented as IC_50_ values. IC_50_ values were determined from a plot presenting inhibitor concentration versus percentage of the enzymatic activity measured as absorbance with *p*NPP substrate of recombinant YopH after 15 min incubation with inhibitors, at 2 mM substrate concentration equal to *Km* value, (n = 3), mean ± SD. **c** The YopH activity after treatment with 50 µM ECR and 50 µM pararosaniline in presence and in absence of catalase (0.3 mg/mL). Data presented as a percent of control, mean ± SD (n = 3). *T*-test analysis of variance, *significantly different from control (*p* < 0.001), **significantly different in pairs (*p* < 0.001)

To indicate the binding affinities of ATA analogues to YopH, we calculated the binding free energy for the complex. The binding affinity indicates that eriochrome cyanine R exhibits stronger binding to YopH than pararosaniline. Figure 4a shows the binding energies to YopH and their components for eriochrome cyanine R and pararosaniline. The components are described below:

- $\Delta E_{vdw}$ = Van der Waals contribution from MM.
- $\Delta E_{ele}$ = Electrostatic energy as calculated by the MM force field.
- $\Delta G_{polar}$ = The electrostatic contribution to the solvation free energy calculated by PB or GB respectively.
- $\Delta G_{non - polar}$ = Nonpolar contribution to the solvation free energy calculated by an empirical model.
- $\Delta G_{bind}$ = Final estimated binding free energy calculated from the terms above (kCal/mol).

$$\Delta G_{bind } = G_{Complex} - G_{{\text{Re} ceptor }} - G_{Ligand}$$

$$\Delta G_{bind } = \Delta E_{MM} + \Delta G_{Polar } + \Delta G_{non - Polar } - T\Delta S$$

$$E_{MM} = E_{\text{int}} + E_{ele} + E_{vdw}$$

Since this is a single trajectory approach, internal energy $E_{\text{int}}$ will be cancelled, so

$$E_{MM} = E_{ele} + E_{vdw}$$

### The inhibitory effect of ATA analogues on YopH phosphatase

In order to assess the inhibitory effect of eriochrome cyanine R and pararosaniline on YopH phosphatase we performed an enzymatic activity assay of recombinant YopH after treatment and 15 min incubation with tested compounds. We calculated IC_50_ values based on a plot presenting ATA analogues concentration versus percentage of the enzymatic activity of recombinant YopH measured as absorbance with *p*NPP substrate. The *p*NPP concentration for IC_50_ calculations was 2 mM equal to *Km* value determined for YopH, where *Km* value is defined as substrate concentration at which enzyme activity is at half maximal. We have found that ECR and pararosaniline are able to decrease the enzymatic activity of YopH in micromolar ranges (Fig. 4b). However, the inhibitory effects of tested compounds were significantly weaker than previously published for ATA, with IC_50_ values in nanomolar ranges (Liang et al. 2003; Kuban-Jankowska et al. 2015). We also found that ECR and pararosaniline inactivate YopH phosphatase reversibly, as the incubation with dithiothreitol of previously inactivated YopH by tested ATA analogues restores the enzyme activity. The reversibility of the ATA analogues induced inactivation was determined with the reduction assay by 30 min incubation with 10 mM DTT.

Based on YopH activity analysis with different substrate *p*NPP concentrations we calculate binding affinity (*Ki*) of the ATA analogues. The obtained *Ki* values for the inhibition of YopH were, for ECR 39.3 ± 2.3 µM and pararosaniline 52.7 ± 2.2 µM, which correspondence to *Ki* = IC_50_/2 for reversible and competitive inhibitors, if the substrate concentration [S] is equal to *Km*.

Furthermore, we have assessed the enzymatic activity of YopH after treatment with ATA analogues in presence and in absence of 0.3 mg/mL catalase. We found that the pretreatment with catalase completely prevents from ATA analogues induced inactivation of YopH (Fig. 4c), the same as we previously observed for ATA (Kuban-Jankowska et al. 2015).

### Viability of macrophage cell line

**Fig. 5:**
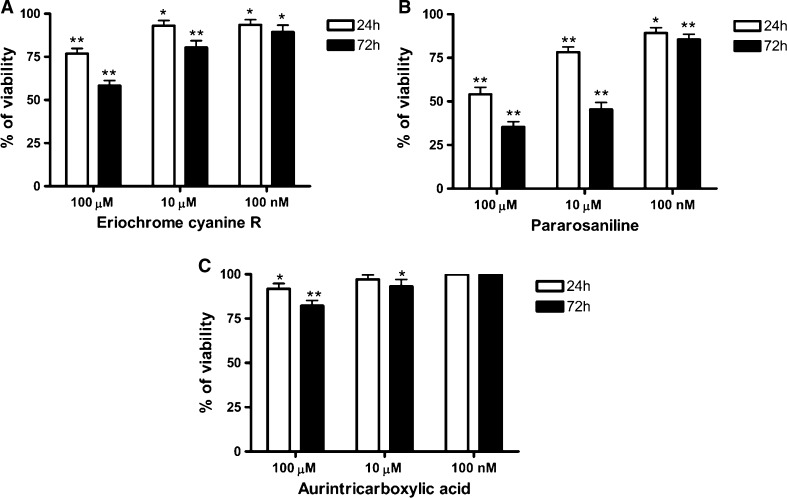
The viability assay of macrophage cells treated for 24 and 72 h with ATA analogous: **a** eriochrome cyanine R, **b** pararosaniline, and **c** ATA . Data presented as percent of the control viability (100 %, cells not treated), mean ± SD (n = 3). One-way Anova test. *Means were significantly different from control (*p* < 0.05). **Means were significantly different from control (*p* < 0.001)

To assess the toxicity of the tested compounds we performed a viability test of macrophage cell line and the results are presented in Fig. 5. The cells were treated for 24 and 72 h with ATA and its analogues: eriochrome cyanine R (Fig. 5a) and pararosaniline (Fig. 5b). We have found that both compounds ECR and pararosaniline decrease the viability of macrophage cells significantly stronger than ATA (Fig. 5c), and that pararosaniline exhibits a stronger effect than ECR.

## Discussion

Aurintricarboxylic acid was characterized as one of the most effective inhibitors of YopH phosphatase, with the IC_50_ values in nanomolar ranges of concentration (Liang et al. 2003; Kuban-Jankowska et al. 2015) and may be considered as a candidate in a drug design studies for YopH virulence factor involved infections. Here, we report the results of the studies we performed for similar compounds, such as eriochrome cyanine R and pararosaniline (Fig. 1b, c) and compare it with our previous findings on inhibitory properties of ATA. Our purpose was to determine how the structural similarity of ATA analogues will have an effect on the ability to inhibit the YopH protein tyrosine phosphatase from *Yersinia enterocolitica*. We selected eriochrome cyanine R and pararosaniline as they possess different functional groups than ATA (Fig. 1a). ECR is more similar to ATA because containing two carboxyl groups, but it also has two methyl groups which change the electron density of the compound. The presence of nitrogen in a pararosaniline structure affects the electron location. The structural changes alter the reactivity of selected compounds and influence the different level of ROS generation.

We performed computational docking and molecular dynamics studies to gain a molecular-level insight into the binding affinities and interactions of ATA analogues in the YopH active site (Figs. 2, 3). The docking analysis confirmed the possibility of binding of ECR and pararosaniline in the YopH catalytic centre. All of the obtained binding conformations were localized to the YopH active site. We have calculated the binding free energy of YopH complex with selected compounds (Fig. 4a). The obtained results showed that ECR possesses a higher binding affinity to the YopH catalytic site than pararosaniline. The computational results were confirmed by the experimental studies of YopH enzymatic activity inhibition. The calculated IC_50_ values indicate that ECR decreases the enzymatic activity of YopH stronger than pararosaniline (Fig. 4b). We conclude that ECR has a stronger binding affinity and inhibitory properties than pararosaniline, as a result of containing carboxyl groups, since the structure of ECR is more similar to ATA than pararosaniline. The presence of catalase prevents from ATA analogues induced inactivation of YopH, as we previously observed for ATA (Kuban-Jankowska et al. 2015). The effect of catalase induced elimination of ATA analogues inhibitory properties allows us to assume that the inactivation mechanism probably involves the oxidation of catalytic cysteine residue, as catalase is an enzyme protecting cells from ROS by decomposition of hydrogen peroxide (Chelikani et al. 2004).

The members of the PTP family, including bacterial YopH phosphatase, share a common catalytic mechanism and a conserved active site motif with a highly reactive nucleophilic Cys residue (Cys403 in YopH), which displays an unusually low pKa of 5 and is situated at the bottom of the pTyr-binding pocket (Sun et al. 2003). In the catalytic mechanism, the active site cysteine initiates a nucleophilic attack on the phosphorus from the substrate and leads to the formation of a thiophosphoryl enzyme intermediate. This process is assisted by the general acid (Asp356 in YopH). The active site arginine (Arg409 in YopH) is involved in initial binding of the pTyr substrate and stabilizes the transition state (Kumar et al. 2004). The molecular dynamics studies showed possible interactions of ATA analogues with residues, which are essential for enzyme activity. We found that eriochrome cyanine R would be able to interact in the active site of YopH with all the essential residues: catalytic cysteine (Cys403), Arg409 residue and general acid (Asp356). The studies of the best binding pose of pararosaniline in the YopH binding site allowed us to observe possible interactions of pararosaniline with general acid residue (Asp356).

Additionally, the cell culture studies showed that pararosaniline has a stronger effect on macrophage cells, reducing their viability more significantly than ECR (Fig. 5a, b). Both compounds were found to possess a significantly higher effect on macrophage cells viability than ATA (Fig. 5c), which reveals only slight impact on viability of cells.

ATA is one of the strongest known YopH inhibitors characterized to date. Here, we show that ATA analogues, eriochrome cyanine R and pararosaniline, can bind effectively into the YopH catalytic site and reversibly reduce the enzymatic activity of YopH. However, they possess weaker inhibitory properties than ATA. The pretreatment with catalase prevents from ATA analogues induced inactivation of YopH, which leads to the conclusion that the inhibition is probably due to the same oxidative mechanism as observed for ATA. The lower level of YopH inactivation, in comparison to ATA, is probably due to the structural changes of ATA analogues which alter their reactivity and influence the lower level of ROS generation than observed for ATA. Computational and experimental results we report here indicate that ECR has stronger binding affinity to YopH and inhibitory properties than pararosaniline, which can be caused by its greater similarity to the structure of ATA. We show also that the tested compounds, especially pararosaniline, significantly decrease the viability of macrophage cells, while ATA is the least cytotoxic of the tested compounds.
